# Association of ACE2 polymorphisms with susceptibility to essential hypertension and dyslipidemia in Xinjiang, China

**DOI:** 10.1186/s12944-018-0890-6

**Published:** 2018-10-20

**Authors:** Yizhi Pan, Tianyi Wang, Yanfang Li, Tianwang Guan, Yanxian Lai, Yan Shen, Abudurexiti Zeyaweiding, Tutiguli Maimaiti, Fang Li, Haiyan Zhao, Cheng Liu

**Affiliations:** 1 Department of Cardiology, Guangzhou First People’s Hospital, Medical School, South China University of Technology, #1 Panfu road, Guangzhou, 510180 China; 2 Department of Cardiology, Guangzhou First People’s Hospital, Guangzhou Medical University, Guangzhou, 510180 China; 3 Department of Cardiology, Shufu People’s Hospital, Kashgar region, 844100 Xinjiang Uygur Autonomous Region (XUAR) China

**Keywords:** Association, ACE2 polymorphism, Essential hypertension, Dyslipidemia, Ischemic stroke

## Abstract

**Background:**

Cardiovascular benefits by reversing environmental risks factors for essential hypertension (EH) and dyslipidemia could be weaken by high genetic risk. We investigated possible associations between ACE2 polymorphisms and dyslipidemia in patients with EH.

**Methods:**

Four hundred and two hypertensive patients were enrolled in an EH group and 233 normotensive individuals were enrolled as control group from the Xinjiang region of China. Fourteen ACE2 polymorphisms were genotyped by Matrix-assisted laser desorption ionization time-of-flight mass spectrometry.

**Results:**

Participants carrying T allele (TT + CT) of rs2074192 (*P* = 0.006), rs4646155 (*P* = 0.030) and rs4646188 (*P* < 0.001), C allele (CT + CT or CC + CG) of rs4240157 (*P* = 0.012), rs4830542 (*P* = 0.020) and rs879922 (P < 0.001) and TT genotype of rs2106809 (*P* = 0.012) were associated with EH. Meanwhile,ACE2 SNPs also exhibited association with dyslipidemia but exhibited obvious heterogeneity. rs1978124 (TT + CT, *P* = 0.009), rs2106809 (TT, *P* = 0.045), rs233575 (CC + CT, *P* = 0.018), rs4646188 (CC, *P* = 0.011) and rs879922 (CC + CG, *P* = 0.003) were association with increased LDL-C (≥1.8 mmol/L). rs2106809 (CC + CT, P < 0.001), rs2285666(TT + CT, *P* = 0.017), rs4646142(CC + CG, *P* = 0.044), rs4646155(TT + CT, P < 0.001) and rs4646188(TT + CT, *P* = 0.033) were association with decreased HDL-C (< 1.0 mmol/L). rs2074192 (TT + CT, P = 0.012), rs4240157 (CC + CT, *P* = 0.027), rs4646156 (AA+AT, *P* = 0.007), rs4646188 (TT + CT, *P* = 0.005), rs4830542 (CC + CT, *P* = 0.047) and rs879922 (CC + CG, *P* = 0.001) were association with increased TC (≥5.2 mmol/L). rs2106809 (*P* = 0.034) and rs4646188 (*P* = 0.013) were associated with hypertriglyceridemia. Further, ischemic stroke was more prevalent with rs4240157 (CC + CT, *P* = 0.043), rs4646188 (CC + CT, P = 0.013) and rs4830542 (CC + CT, *P* = 0.037). In addition, rs2048683 and rs6632677 were not association with EH, dyslipidemia and ischemic stroke.

**Conclusion:**

The ACE2 rs4646188 variant may be a potential and optimal genetic susceptibility marker for EH, dyslipidemia and its related ischemic stroke.

**Electronic supplementary material:**

The online version of this article (10.1186/s12944-018-0890-6) contains supplementary material, which is available to authorized users.

## Background

Essential hypertension (EH) is a clinical syndrome characterized by increased systemic arterial pressure (SBP ≥ 140 mmHg/ DBP ≥ 90 mmHg) that often leads to dysfunction or damage of organs including the heart, brain and kidney. The incidence of hypertension has steadily increased over the past decade [1], and the proportion of hypertensive patients with other cardiovascular risk factors (e.g., dyslipidemia, overweight/obesity and lack of physical activity, etc) has increased continuously with the increasing prevalence of EH in China [2]. Hypertension plus dyslipidemia is also recognized as the leading cause in global death of vascular disease [3]. Genetic background notwithstanding, comprehensive management of those multiple modifiable risk factors (e.g., unhealthy diet, lack of physical activity, smoking, obesity, and dyslipidemia, etc) is significant associated with lower blood pressure, lower LDL-C and lower cardiovascular events (e.g., arteriosclerosis cardiovascular disease (ASCVD)) [4], but the cardiovascular benefits of healthy lifestyles could be weaken or offset by high genetic risk [5]. Thus, clinical evaluation of genetic background must be considered along with emphasizing the influence of lifestyle modification on the prevention of hypertension, dyslipidemia and its related cardiovascular events [6].

Numerous candidate genes have been implicated in susceptibility to EH. In recent years genes of the renin-angiotensin-aldosterone system (RAAS) have received a good deal of attention. Angiotensin converting enzyme 2 (ACE2) is an important regulator of RAAS (a homolog of ACE), and a monocarboxypeptidase that converts angiotensin II (Ang II) into angiotensin 1–7 (Ang 1–7) which, by virtue of its actions on the Mas receptor, negatively regulates Ang II-induced cardiovascular damage, and exhibites notable cardiovascular protective effects [7]. ACE2 maps to chromosome Xp22, spans 39.98 kb of genomic DNA, and contains 20 introns and 18 exons. The ACE2 gene encodes a type I membrane-bounding glycoprotein composed by 805 amino acids. Functional domains include a C-terminal transmembrane anchoring region, N-terminal signal peptide region and an HEXXH zinc binding metalloprotease motif. ACE2 polymorphisms not only associated with EH in the Chinese population [8] but also exhibited geographical (southern vs. northern [9–12]), ethnic (Han vs. non-Han nationality) [11–13], and gender (females and males [9, 10]) diversity. However, the association of ACE2 SNPs with dyslipidemia and ASCVD (e.g., ischemic stroke (IS)) in Xinjiang region of China are rarely reported. Theoretically, there may be common genetic basis between dyslipidemia, hypertension and its related IS [14, 15] manifesting the characteristics of ethnic-specific genetic pleiotropy [16]. In this study we investigated possible associations of ACE2 gene variations with hypertension, dyslipidemia and its related cardiovascular events in Xinjiang.

## Methods

### Study participants

This study was reviewed and approved by the Ethics Committee of Guangzhou First People’s Hospital, the Second Affiliated Hospital of South China University of Technology. From August 2012 to December 2017, a total of 402 consecutive patients with EH (222 Han and 180 Uygur) and 233 normotensive subjects (116 Han and 117 Uygur) from the southern Xinjiang, China were enrolled in the study. Both Han and Uygur participants were long resident in the region and were from multi-generation resident families. The newly hypertensive patients were diagnosed according to the criteria of the 1999 World Health Organization/International Society of Hypertension (WHO/ISH) as follows: (1) systolic blood pressure (SBP) ≥ 140 mmHg and/or diastolic blood pressure (DBP) ≥ 90 mmHg; (2) diagnosed as EH for the first time and did not receive any antihypertensive treatment. Any participants diagnosed with white coat hypertension and secondary hypertension were excluded from the study according to 2013 ESH/ESC guidelines for the management of arterial hypertension [17]. The normotensive individuals were recruited a medical examination at the same hospital, and were clinically confirmed in the absence of hypertension according to previously described methods with slight modifications [18]. All stroke participants were survivors of ischemic stroke (IS), and diagnosed by computed tomography and/or magnetic resonance image scanning of the brain according to guidelines for prevention of stroke in patients with ischemic stroke or transient ischemic attack [19]. All biochemistry tests were performed by standard methods in the Chemical Laboratory.

### Genotyping assay

Genomic DNA was extracted from whole blood using the Maxwell RSC Whole blood DNA kit (Promega, Madison, WI), quantified using NanoDrop-1000 (ThermoFisher, Waltham, MA) and diluted to 10 ng/μL concentration. Fourteen ACE2 SNPs (rs1978124, rs2048683, rs2074192, rs2106809, rs2285666, rs233575, rs4240157, rs4646142, rs4646155, rs4646156, rs4646188, rs4830542, rs6632677 and rs879922) were identified based on existing literature and human genome sequence databases. Primers for ACE2 SNPs were designed based upon sequence information from GenBank using Primer 5.0 (Whitehead Institute Cambridge, Massachusetts, USA) and Operon’s Oligo software 7.60 (OperonTechnologies Inc., Alameda, California, USA). Primers are shown in Additional file 1: Table S1. ACE2 SNPs were analyzed using the Sequenom MassARRAY system according to previously described methods [20]. Genotyping accuracy was determined by genotype concordance between duplicate samples and was 100% for each SNP.

### Statistical analysis

Because ACE2 is located on the X chromosome, Hardy–Weinberg equilibrium was assessed only for females as shown in Additional file 1: Table S2. Analysis was performed using SPSS version 20 (SPSS, Chicago, IL). Categorical variables (gender, nationality, EH, high low-density lipoprotein cholesterol(LDL-C ≥ 1.8 mmol/L), low high-density lipoprotein cholesterol (HDL-C <  1.0 mmol/L), high total cholesterol (TC ≥ 5.2 mmol/L), high triglyceridemia (TRIG≥1.7 mmol/L) [21] and IS) were presented as frequencies. The relationship between each ACE2 SNP and those categorical variables were assessed using the Chi square test. The Odds ratio (OR) between control genotype and high hypertensive risk genotype for each ACE2 SNP among categorical variables was evaluated using binary logistic regression. Considering the possible false positive risk to the final result, Bonferroni adjustment was applied to adjust the *p*-value obtained in multi-logit regression. Continuous variables (age, SBP, DBP, BMI and blood biochemical index) were presented as mean ± SD. Significant differences for continuous variables were analyzed by two/one way ANOVA or independent-sample t-test according to our research design. The least significant difference (LSD) test was further used to assess differences for two subgroups after variance analysis, to show distinct differences with homogeneous variance, while the Games-Howell test was used for heterogeneous variance. A *P* value less than 0.05 was considered statistically significant. All probabilities are two-tailed.

## Result

### Characteristics of the study participants

Among both Han and Uygur participants, hypertensive and normotensive subjects showed significant differences in SBP, DBP, BMI, LDL-C, serum sodium, serum uric acid, HsCRP and the activation of RAAS (all *P* < 0.05) but not in gender, age, smoking, drinking, TRIG, TC, HDL-C, lipoprotein A, blood glucose, renal function (Cr, BUN), liver function (ALT, AST, Alb) and blood electrolytes (calcium, potassium and magnesium) (all *P* > 0.05) (see Additional file 1: Table S3).

### Association of ACE2 SNPs and EH

As shown in Table 1, ACE2 SNPs rs2074192 (*P* = 0.006), rs2106809 (*P* = 0.012), rs4240157 (*P* = 0.012), rs4646155 (*P* = 0.030), rs4646188 (*P* < 0.001), rs4830542 (*P* = 0.020) and rs879922 (P < 0.001) were significantly associated with EH.

**Table 1:** Association of ACE2 SNPs with EH in participants

ACE2 SNPs		Normotensive	Hypertensive	OR(95%CI)^a^	*P*-value^a^
		(N/%)	(N/%)		
*rs2074192*	*CC*	118 (50.6)	162(40.3)	1.00	
	*TT + CT*	115 (59.4)	240(59.7)	1.72 (1.17–2.53)	0.006
*rs2106809*	*CC + CT*	121 (51.9)	175(43.5)	1.00	
	*TT*	112(48.1)	227(56.5)	1.71 (1.13–2.58)	0.012
*rs4240157*	*CC + CT*	43 (18.5)	92 (22.9)	1.99 (1.17–3.41)	0.012
	*TT*	190 (81.5)	310 (77.1)	1.00	
*rs4646155*	*CC*	211 (90.6)	334 (83.1)	1.00	
	*TT + CT*	22 (9.4)	68 (16.9)	1.94 (1.06–3.54)	0.030
*rs4646188*	*CC*	74 (31.8)	85 (21.1)	1.00	
	*TT + CT*	159 (68.2)	317 (78.9)	3.25 (1.95–5.41)	< 0.001
*rs4830542*	*CC + CT*	43 (18.5)	90 (22.4)	1.88 (1.10–3.23)	0.020
	*TT*	190 (81.5)	312 (77.6)	1.00	
*rs879922*	*CC + CG*	40 (17.2)	131 (32.6)	4.86 (2.74–8.64)	< 0.001
	*GG*	193 (82.8)	271 (67.4)	1.00	

^a^After adjustment for nationality, gender, age, smoking, BMI, TRIG, LDL-C, HDL-C, Lp(a), FBS, UA, HsCRP and Ang II

### Association of ACE2 SNPs with increased LDL-C (≥ 1.8 mmol/L)

As shown in Table 2, ACE2 SNPs rs1978124 (*P* = 0.009), rs2236306 (*P* = 0.045), rs233575 (*P* = 0.018), rs4646188 (*P* = 0.011) and rs879922 (*P* = 0.003) were associated with high low-density lipoprotein cholesterol.

**Table 2:** Association of ACE2 SNPs with increased LDL-C (≥1.8 mmol/L) in study subjects

ACE2 SNPs		LDL-C (N/%)		OR(95%CI)^a^	*P*-value^a^
		< 1.8 mmol/L	≥1.8 mmol/L		
*rs1978124*	*CC*	122 (91.0)	405 (80.0)	1.00	
	*TT + CT*	12 (9.0)	96 (19.2)	2.51 (1.26–5.01)	0.009
*rs2106809*	*CC + CT*	70 (52.2)	226 (45.1)	1.00	
	*TT*	64 (47.8)	275 (54.9)	1.58 (1.01–2.46)	0.045
*rs233575*	*CC + CT*	9 (6.7)	75 (15.0)	2.52 (1.17–5.44)	0.018
	*TT*	125 (93.3)	426 (85.0)	1.00	
*rs4646188*	*CC*	30 (22.4)	129 (25.7)	1.96 (1.16–3.29)	0.011
	*TT + CT*	104 (77.6)	372 (74.3)	1.00	
*rs879922*	*CC + CG*	20 (14.9)	151 (30.1)	2.48 (1.35–4.55)	0.003
	*GG*	114 (85.1)	350 (69.9)	1.00	

^a^After adjustment for nationality, gender, age, BMI, EH, FBS, HsCRP and Ang II

### Association of ACE2 SNPs with decreased HDL-C (< 1.0 mmol/L)

As shown in Table 3, ACE2 SNPs rs2106809 (*P* < 0.001), rs2285666 (*P* = 0.017), rs4646142 (*P* = 0.044), rs4646155 (*P* < 0.001) and rs4646188 (*P* = 0.033) were significantly associated with low high-density lipoprotein cholesterol.

**Table 3:** Association of ACE2 SNPs with decreased HDL-C (**<** 1.0 mmol/L) in study subjects

ACE2 SNPs		HDL-C (N/%)		OR(95%CI)^a^	*P*-value^a^
		≥ 1.0 mmol/L	< 1.0 mmol/L		
*rs2106809*	*CC + CT*	215 (42.1)	81 (65.3)	2.49 (1.58–3.91)	< 0.001
	*TT*	296 (57.9)	43 (34.7)	1.00	
*rs2285666*	*CC*	194 (38.0)	37 (29.8)	1.00	
	*TT + CT*	317 (62.0)	87 (70.2)	1.73 (1.10–2.71)	0.017
*rs4646142*	*CC + CG*	318 (62.2)	85 (68.5)	1.58 (1.01–2.46)	0.044
	*GG*	193 (37.8)	39 (31.5)	1.00	
*rs4646155*	*CC*	453 (88.6)	92 (74.2)	1.00	
	*TT + CT*	58 (11.4)	32 (25.8)	2.85 (1.71–4.75)	< 0.001
*rs4646188*	*CC*	123 (24.1)	36 (29.0)	1.00	
	*TT + CT*	388 (75.9)	88 (71.0)	1.88 (1.05–3.35)	0.033

^a^After adjustment for nationality, gender, age, BMI, EH, FBS, HsCRP and Ang II

### Association of ACE2 SNPs with increased TC (≥5.2 mmol/L)

As shown in Table 4, ACE2 SNPs rs2074192 (*P* = 0.012), rs4240157 (*P* = 0.027), rs4646156 (*P* = 0.007), rs4646188 (*P* = 0.005), rs4830542 (*P* = 0.047) and rs879922 (*P* = 0.001) were associated with high TC.

**Table 4:** Association of ACE2 SNPs with increased TC (**≥** 5.2 mmol/L) in study subjects

ACE2 SNPs		TC (N/%)		OR(95%CI)^a^	*P*-value^a^
		< 5.2 mmol/L	≥5.2 mmol/L		
*rs2074192*	*CC*	240 (46.0)	40 (35.4)	1.00	
	*TT + CT*	282 (54.0)	73 (64.6)	1.83 (1.14–2.94)	0.012
*rs4240157*	*CC + CT*	102 (19.5)	33 (29.2)	1.78 (1.07–2.98)	0.027
	*TT*	420 (80.5)	80 (70.8)	1.00	
*rs4646156*	*AA + AT*	57 (10.9)	21 (18.6)	2.34 (1.07–4.32)	0.007
	*TT*	465 (89.1)	92 (81.4)	1.00	
*rs4646188*	*CC*	143 (27.4)	16 (14.2)	1.00	
	*TT + CT*	379 (72.6)	97 (85.8)	2.31 (1.29–4.13)	0.005
*rs4830542*	*CC + CT*	100 (19.2)	33 (29.2)	1.64 (1.01–2.66)	0.047
	*GT*	422 (80.8)	80 (70.8)	1.00	
*rs879922*	*CC + CG*	123 (23.6)	48 (42.5)	2.17 (1.38–3.41)	0.001
	*GG*	399 (76.4)	65 (57.5)	1.00	

^a^After adjustment for nationality, gender, age, BMI, EH, FBS, HsCRP and Ang II

### Association of ACE2 SNPs with increased TRIG (≥1.7 mmol/L)

As shown in Table 5, ACE2 SNPs rs2106809 (*P* = 0.034) and rs4646188 (*P* = 0.013) were associated with hypertriglyceridemia.

**Table 5:** Association of ACE2 SNPs with increased TRIG (≥1.7 mmol/L) in study subjects

ACE2 SNPs		TRIG (N/%)		OR(95%CI)^a^	*P*-value^a^
		< 1.7 mmol/L	≥1.7 mmol/L		
*rs2106809*	*CC + CT*	229(45.6)	67(50.4)	1.61(1.04–2.49)	0.034
	*TT*	273(54.4)	66(49.6)	1.00	
*rs4646188*	*CC*	120(23.9)	39(29.3)	1.80(1.13–2.85)	0.013
	*TT + CT*	382(76.1)	94 (70.7)	1.00	

^a^After adjustment for nationality, gender, age, BMI, EH, FBS, HsCRP and Ang II

### Association of ACE2 SNPs with ischemic stroke

As shown in Table 6, ACE2 SNPs rs4240157 (*P* = 0.043), rs4646188 (*P* = 0.013) and rs4830542 (*P* = 0.037) were associated with ischemic stroke.

**Table 6:** Association of ACE2 SNPs with ischemic stroke in study subjects

ACE2 SNPs		Non-IS	IS	OR(95%CI)^a^	*P*-value^a^
		(N/%)	(N/%)		
*rs4240157*	*CC + CT*	114 (20.2)	21 (30.0)	2.18 (1.03–4.65)	0.043
	*TT*	451 (79.8)	49 (70.0)	1.00	
*rs4646188*	*CC + CT*	135 (23.9)	24 (34.3)	2.44 (1.21–4.91)	0.013
	*TT*	430 (76.1)	46 (65.7)	1.00	
*rs4830542*	*CC + CT*	112 (19.8)	21 (30.0)	2.25 (1.05–4.80)	0.037
	*TT*	453 (80.2)	49 (70.0)	1.00	

^a^After adjustment for nationality, gender, age, smoking, BMI, SBP, DBP, LDL-C, HDL-C, FBS, HsCRP and Ang II

## Discussion

EH and dyslipidemia is rapidly developing into an epidemic, and dramatically increases the global cardiovascular events that have become a serious public health problem especially in developing countries (e.g., China) [2], which has become the leading cause of ASCVD-related death (e.g., IS) during the last decade in China [22]. The treatment and prevention outlook for EH and dyslipidemia is more severe in China [23], especially in minority areas (e.g., Xinjiang) owing to the prevalence of EH and dyslipidemia differs among different geographic areas (urban and rural) and ethnicities (Han and non-Han (e.g., Uygur) populations) while at the same time EH and dyslipidemia related stroke is extremely high [16, 24]. Thus, early identification and assessing populations at high risk of EH and dyslipidemia are the key steps in ASCVD (e.g., IS) prevention and control.

This study addressed possible relationships of ACE2 polymorphisms with EH and dyslipidemia in south Xinjiang region of China. We found that the genotypes of rs2074192 (TT + CT), rs2106809 (TT), rs4240157 (CC + CT), rs4646155 (TT + CT) and rs4830542 (CC + CT) were linked to moderate EH risk (OR = 1.71–1.99) while rs4646188 (TT + CT) and rs879922 (CC + CG) linked to high EH risk (OR = 3.25–4.86). Our findings are consistent with the observation by Patel et al. [25] who reported ACE2 SNPs (rs2074192, rs4240157 and rs4646188) were associated with higher hypertension risk while rs1978124 was not in a diabetic Australian Caucasian population, and the study by Benjafield et al. [26] who reported that rs1978124 was not correlated with EH in Australian persons of Anglo-Celtic descent. In this study we newly found rs4646155 and rs879922 were association with EH, but our results are in contrast to the observations by Niu W et al. [9]. The association of ACE2 SNP rs2285666 with hypertension exhibit high genetic heterogeneity and varies with geographical, ethnic and gender [27], this loci was not linked to hypertension in Xinjiang, which was consistent with previously reported the association in northwestern [11] and central [12] China. In addition, this is the first report showing that SNP rs4830542 was associated with EH while rs2048683 was not correlated with EH (see Additional file 1: Table S4).

Dyslipidemia is the second leading cause of cardiovascular disease-related death after hypertension [23]. The prevalence of hypertension plus dyslipidemia and overweight/obesity was reportedly high in Xinjiang [16, 28] and we made similar observations in the course of this study. Indeed, we also observed that the levels of BMI and LDL-C in hypertensive patients were close to levels of hypertensive patients in the United States [29]. To the best of our knowledge, this is the first more comprehensive study to investigate the association of ACE2 gene polymorphism with dyslipidemia. In previous studies SNP rs2285666 was not linked to dyslipidemia, but its relationship with various subtypes of dyslipidemia is not shown [30]. In this study we found that 9 ACE2 polymorphic loci were respectively correlated with only one type of dyslipidemia, including higher LDL-C (rs1978124 and rs233575), lower HDL-C (rs2285666, rs4646142 and rs4646155) and higher TC (rs2074192, rs4240157, rs4646156 and rs4830542). ACE2 SNP rs879922 (LDL-C and TC), rs2106809 (LDL-C, HDL-C and TRIG) and rs4646188 (LDL-C, HDL-C, TC and TRIG) were correlated with more than 2 types of dyslipidemia. However, 2 ACE2 polymorphic loci (rs2048683 and rs6632677) were non-correlated with any type of dyslipidemia (see Additional file 1: Table S5-S8). Not all dyslipidemia risk related ACE2 variation was also associated with hypertension (e.g., rs1978124, rs2285666, rs233575, rs4646142 and rs4646156), but all 7 EH risk related variation were also significantly correlated with dyslipidemia. Almost all high hypertensive risk genotypes of all EH risk related variations exhibited association with moderate to high risk of dyslipidemia except rs2106809 and rs4646188. Particularly, patients carrying the high EH risk genotype (TT) of rs2106809 were associated with increased LDL-C but those carrying the control genotype (CC + TT) of this loci was associated with decreased HDL-C and hypertriglyceridemia. By contrast, patients carrying the control genotype (CC) of rs4646188 were associated with increased LDL-C and TRIG but those carrying high EH risk genotype (TT + CT) of this loci was associated with the other two types of dyslipidemia. ACE2 polymorphisms correlations with elevated risk of dyslipidemia were obvious heterogeneity in Xinjiang.

Both hypertension and dyslipidemia are clinical risk factors for ASCVD, and the risk for ASCVD increases following the increase in blood pressure and blood lipid level [31]. In this study we found that 3 ACE2 SNPs (rs1978124, rs2074192 and rs879922) were linked to moderate and high risk of EH or dyslipidemia (increased LDL-C and TC), which previously reported that the three loci were associated with cardiovascular death [32], suggesting that there is a common genetic basis for hypertension, dyslipidemia and cardiovascular events [33, 34]. Despite EH patients with dyslipidemia received standard hypotensive therapy and lipid-lowering therapy, there is still a significant increase residual risk of ASCVD, that is related to atherogenic dyslipidemia (high TRIG and low HDL-C level) [35]. Our results showed that 2 SNPs (rs2285666 and rs4646142) was not associated with EH (see Additional file 1: Table S4) but exhibited association with decreased HDL-C, which were consistent with previously reported association of the two loci with ASCVD (e.g., coronary heart disease [36], IS [37]). In this study we newly found that ACE2 SNPs rs4646188 were not only correlated with hypertension and atherogenic dyslipidemia but also linked to high risk of ischemic stroke. Four ACE2 SNPs (rs2074192, rs4240157, rs4830542 and rs879922) were association with hypertension and increased TC, but only rs4240157 and rs4830542 also exhibited association with high stroke risk in our study. Meanwhile, we found that rs2074192 and rs879922 were not only non-correlated with IS but also had nothing to do with the recurrence risk of stroke [38]. Although rs6632677 was not associated with hypertension, dyslipidemia and stroke, it was found to be related to left ventricular remodeling [39] and possible atrial fibrillation risk in Chinese population [40]. However, association of SNP rs2048683 with hypertension, dyslipidemia and stroke had been pretty “silent”, suggesting that the loci were not protective factors but they are at least not a harmful factor on EH and EH related cardiovascular events.

Indeed, the RAAS activation plays a key role in the occurrence and progression of hypertension, dyslipidemia and its related ASCVD (e.g., IS). The circulation and tissue RAAS of hypertension with dyslipidemia are excessively activated [41], which promotes the accumulation of ox-LDL in the blood vessels and further accelerates the process of atherosclerosis. On the other hand, disequilibrium of vascular lipid homeostasis (especially the accumulation of ox-LDL) enhances the activation of RAAS [42]. ACE2 is an essential regulator by antagonizing Ang II mediated cardiovascular injury. Although the roles of ACE2 gene polymorphisms (mutations or variants) on hypertension, dyslipidemia and its related ASCVD were incompletely understood, it may be related to the cross-talk between ACE2/Ang-(1–7)/Mas axis and ACE/Ang II/AT1 axis [43]. ACE2 gene polymorphisms (e.g., rs2106809 [44], rs2074192 [43]) were associated with downregulation of circulating Ang-(1–7). The deletion of ACE2 in mice model was associated with increased circulation and tissue Ang II levels [45], led to cardiovascular damage [46]. The possible mechanism would be related to changes in quantity and function of ACE2 mutant protein owing to ACE2 polymorphism related amino acid substitutions(e.g., 1075A/G(rs1978124), 8790G/A (rs2285666) and 16854G/C(rs4646142), etc. [25, 47]), which might be involved in the posttranscriptional regulation via microRNA, the mRNA splicing efficiency (e.g., intron splicing enhancer or silencer element) and mRNA stability (e.g., conformation) of ACE2, at least partly confirmed by recent research that the changes of ACE2 expression was in protein level rather than mRNA level in mice [48], and microRNA might regulate RAAS activity via by altering the interaction between microRNA and mRNA of targeted gene [44]. In addition, ACE2 SNP rs4830542 was found to be located in the 3’-UTR region while the other 13 ACE2 SNPs (e.g., rs4646188) were in intron. Regardless of those SNPs were in 3’UTR region or intron (both in noncoding region of ACE2 gene), it is still unknown which SNPs is the functional SNP. Potentially, ACE2 SNP rs4646188 is hopeful to be the functional SNP because it is located in a splicing site of ACE2 gene that needs to be investigated further.

Some limitations should be mentioned. First, since our sample size is not large enough, further prospective large sample studies are needed to validate our findings. Secondly, the possibility of false-positive findings should be considered especially for secondary study based on our results.

## Conclusion

ACE2 SNP rs4646188 may be a potential and optimal genetic susceptibility marker for hypertension, dyslipidemia and its related cardiovascular events (i.e., ischemic stroke). Specially, our data showed for the first time that the ACE2 SNP rs4830542 was associated with hypertension and dyslipidemia. Our observations further support that the genetic predisposition of ACE2 SNPs associated with the risk of EH, dyslipidemia and its related cardiovascular events should need large-scale evaluation as well as in different ethnic groups.

## Additional file

Additional file 1: **Table S1.** ACE2 SNP primers used in the Sequenom MassARRAY system. **Table S2.** Descriptive information on ACE2 SNPs in study participants. **Table S3**. Baseline characteristics of study participants. **Table S4.** Association of 7 ACE2 SNPs with EH in participants. **Table S5.** Association of ACE2 SNPs with increased LDL-C (≥1.8 mmol/L) in study subjects Table S6 Association of ACE2 SNPs with decreased HDL-C (< 1.0 mmol/L) in study subjects. **Table S7.** Association of ACE2 SNPs with increased TC (≥5.2 mmol/L) in study subjects. **Table S8.** Association of ACE2 SNPs with increased TRIG (≥1.7 mmol/L) in study subjects. (DOCX 84 kb)

## Abbreviation

ACE2: Angiotensin converting enzyme 2
Alb: Albumin
ALD: Aldosterone
ALT: Alanine aminotransferase
Ang I/ II: Angiotensin I/II
ASCVD: Arteriosclerosis cardiovascular disease
AST: Aspartate aminotransferase
BMI: Body mass index
BUN: Blood urea nitrogen
Cr: Creatinine
DBP: Diastolic blood pressure
EH: Essential hypertension
FBG: Fasting blood glucose
HbA1C: Glycosylated hemoglobin
HDL-C: High-density lipoprotein cholesterol
HsCRP: High-sensitivity C-reactive protein
IS: Ischemic stroke
LDL-C: Low-density lipoprotein cholesterol
Lp(a): Lipoprotein A
LVEF: Left ventricular ejection fraction
LVMI: Left ventricular mass index
MAF: Linor allele frequency
MAU: Licroalbuminuria
RAAS: Renin-angiotensin-aldosterone system
SBP: Systolic blood pressure
SNP: Single nucleotide polymorphism
T2D: Type 2 diabetes mellitus
TC: Total cholesterol
TRIG: Triglyceridemia
UA: Blood uric acid
