# Supplementary material for: Association of ACE2 polymorphisms with susceptibility to essential hypertension and dyslipidemia in Xinjiang, China
Source: Lipids Health Dis. 2018 Oct 20;17:241. doi: 10.1186/s12944-018-0890-6 (PMC6195726; doi:10.1186/s12944-018-0890-6)
Supplement: Supplementary file 1 — Table S1. ACE2 SNP primers used in the Sequenom MassARRAY system. Table S2. Descriptive information on ACE2 SNPs in study participants. Table S3. Baseline characteristics of study participants. Table S4. Association of 7 ACE2 SNPs with EH in participants. Table S5. Association of ACE2 SNPs with increased LDL-C (≥1.8 mmol/L) in study subjects Table S6 Association of ACE2 SNPs with decreased HDL-C (< 1.0 mmol/L) in study subjects. Table S7. Association of ACE2 SNPs with increased TC (≥5.2 mmol/L) in study subjects. Table S8. Association of ACE2 SNPs with increased TRIG (≥1.7 mmol/L) in study subjects. (DOCX 84 kb) [file 12944_2018_890_MOESM1_ESM.docx]

**Additional file 1：Additional Method and Results**

**Additional Method**

This study was reviewed and approved by the Ethics Committee of Guangzhou First People’s Hospital, the Second Affiliated Hospital of South China University of Technology. From August 2012 to December 2017, a total of 402 consecutive patients with EH (222 Han and 180 Uygur) and 233 normotensive subjects (116 Han and 117 Uygur) from the southern Xinjiang, China were enrolled in the study. Both Han and Uygur participants were long resident in the region and were from multi-generation resident families. The newly hypertensive patients were diagnosed according to the criteria of the 1999 World Health Organization/International Society of Hypertension (WHO/ISH) as follows: (1) systolic blood pressure (SBP) ≥ 140 mmHg and/or diastolic blood pressure (DBP) ≥ 90 mmHg; (2) diagnosed as EH for the first time and did not receive any antihypertensive treatment. Any participants diagnosed with white coat hypertension and secondary hypertension were excluded from the study according to 2013 ESH/ESC guidelines for the management of arterial hypertension [1]. The normotensive individuals were recruited a medical examination at the same hospital, and were clinically confirmed in the absence of hypertension according to previously described methods with slight modifications [2]. Blood pressure was measured in the seated position after 10 min of rest using a mercury sphygmomanometer by experienced and certified examiners, and measured in the brachial artery 3 times at 5-minute intervals in at least two separate visits to the health care office. The mean of the last 2 measurements per visit was recorded as representative of clinic BP. Ischemic stroke (IS) was determined by the presence of a focal or global neurological deficit with symptoms and signs lasting more than 24 h. All stroke participants were survivors of IS, and diagnosed by computed tomography and/or magnetic resonance image scanning of the brain according to guidelines for prevention of stroke in patients with ischemic stroke or transient ischemic attack [3]. All biochemistry tests were performed by standard methods in the Chemical Laboratory. In particular, the plasma components (ACE, renin, ANGI/II and ALD) of RAAS were analyzed using a Maglumi 4000 Automated Chemiluminescence Immunoassay system (Snibe Co., Ltd., Shenzhen, China) according to previously described methods [4].

**Additional Results**

**ACE2 SNPs and genotype frequencies**

Because ACE2 is on the X-chromosome, we assessed Hardy-Weinberg equilibrium only for female subjects. Not all ACE2 SNPs examined were in Hardy-Weinberg equilibrium (P>0.05) and heterogeneity was observed in study participants (see Table S2). In female subjects ACE2 SNP rs2074192, rs2285666, rs4240157, rs4646142, rs4830542 and rs879922 were in Hardy-Weinberg equilibrium (P>0.05).

**Association of ACE2 SNPs and EH**

As shown in Table S4, ACE2 SNPs rs1978124, rs2048683, rs2285666, rs233575, rs4646142, rs4646156 and rs6632677 were not associated with EH(all P>0.05).

**Association of ACE2 SNPs with increased LDL-C (≥1.8mmol/L) in study subjects**

As shown in Table S5, 7 ACE2 SNPs (rs2074192, rs2285666, rs4240157, rs4646142, rs4646155, rs4830542 and rs6632677) were not associated with increased LDL-C (≥1.8mmol/L, all P>0.05). In addition, significant differences were observed between participants with or without LDL-C≥1.8mmol/L for genotype distributions of ACE2 SNP rs2048683 and rs4646156(both P<0.05), but considering the limit of the sample size, the estimation for the potential association between the loci and increased LDL-C (≥1.8mmol/L) may be biased. Therefor the OR for the 2 loci will not be estimated.

**Association of ACE2 SNPs with decreased HDL-C (<1.0mmol/L) in study subjects**

As shown in Table S6, 5 ACE2 SNPs (rs2074192, rs4240157, rs4830542, rs6632677 and rs879922) were not associated with decreased HDL-C (<1.0mmol/L, all P>0.05). The OR of decreased HDL-C risk for 4 loci (rs1978124, rs2048683, rs233575 and rs4646156) will not be estimated due to possible bias.

**Association of ACE2 SNPs with increased TC (≥5.2mmol/L) in study subjects**

As shown in Table S7, 7 ACE2 SNPs (rs1978124, rs2048683, rs2106809, rs2285666, rs233575, rs4646142 and rs6632677) were not associated with increased TC (≥5.2mmol/L, all P>0.05). The OR of increased TC risk for rs4646155 will not be estimated due to possible bias.

**Association of ACE2 SNPs with increased TRIG (≥1.7mmol/L) in study subjects**

As shown in Table S8, 11 ACE2 SNPs (rs1978124, rs2048683, rs2074192, rs2285666, rs233575, rs4240157, rs4646142, rs4646155, rs4646156, rs4830542 and rs879922) were not associated with increased TRIG (≥1.7mmol/L, all P>0.05). The OR of increased TRIG risk for rs6632677 will not be estimated due to possible bias.

**Table legends**

Table S1 ACE2 SNP primers used in the Sequenom MassARRAY system

Table S2 Descriptive information on ACE2 SNPs in study participants

Table S3 Baseline characteristics of study participants

Table S4 Association of 7 ACE2 SNPs with EH in participants

Table S5 Association of ACE2 SNPs with increased LDL-C (≥1.8mmol/L) in study subjects Table S6 Association of ACE2 SNPs with decreased HDL-C (<1.0mmol/L) in study subjects

Table S7 Association of ACE2 SNPs with increased TC (≥5.2mmol/L) in study subjects

Table S8 Association of ACE2 SNPs with increased TRIG (≥1.7mmol/L) in study subjects

Table S1 The primers of ACE2 SNPs in the Sequenom MassARRAY system

| **NQ.** | **SNP_ID** | **2nd-** **reverse PCR primer (5’-3’)** | **1st-** **forward PCR primer (5’-3’)** | **extension primer (5’-3’)** |
| --- | --- | --- | --- | --- |
| 1 | *rs1978124* | *ACGTTGGATGGAGAGAACTTTGGAAACCTG* | *ACGTTGGATGAAGCTGCTGATGTAGAAGTG* | *CCATATCTCTATCTGATGGAC* |
| 2 | *rs2048683* | *ACGTTGGATGCAGTGAACATGGCAGTGTAG* | *ACGTTGGATGTGATCCAGCAATCCCTCTTC* | *GGCAGTGTAGATATCTTTATGAAG* |
| 3 | *rs2074192* | *ACGTTGGATGTTAGGTTCATCAACAGCTCC* | *ACGTTGGATGCCCTTAAACACAGCAGTCAC* | *CAAGGGTGGAAATGTATAAATGGTTGG* |
| 4 | *rs2106809* | *ACGTTGGATGAATAACAGTCTCTCTCCCCC* | *ACGTTGGATGTTCCTGGGTAGATGGCAATG* | *GTTTTCCTGCACACCATCTGAT* |
| 5 | *rs2285666* | *ACGTTGGATGCTGAGAGAAAAGTAAATTTCA* | *ACGTTGGATGCCAGATAATCCACAAGAATGC* | *CATAATCACTACTAAAAATTAGTAGC* |
| 6 | *rs233575* | *ACGTTGGATGAGGTCCTATGACCAAGTCTC* | *ACGTTGGATGTTTCTTATGTGCCTCCCCAG* | *TCCTATGACCAAGTCTCTATAGTA* |
| 7 | *rs4240157* | *ACGTTGGATGTTGCTCAGTGAATTGGCCTC* | *ACGTTGGATGTTTCCATGCAGTGAGGGTTG* | *CTCAGAACATTACAGAATCAAAC* |
| 8 | *rs4646142* | *ACGTTGGATGTTAGGTGAAGCGTTCCTGTC* | *ACGTTGGATGAGCCCATGGACCCAATAAAG* | *GGAGATGGTTGCTTTGTAGTCTC* |
| 9 | *rs4646155* | *ACGTTGGATGGGCATGTTCTTAACCTTGGC* | *ACGTTGGATGCCAATATGACCCTGTAAACC* | *GGATGAACCTTGGCAAAATAAACTT* |
| 10 | *rs4646156* | *ACGTTGGATGGGGAAATAGATATGATGGGC* | *ACGTTGGATGCCTTAGGTACTTGGACCTTC* | *GGGCCATGGAACAGG* |
| 11 | *rs4646188* | *ACGTTGGATGGATATTCTACTCAGAAACG* | *ACGTTGGATGCTCTGTGTTCCCTTCTGTTG* | *GGGGAACGTAGAATTTTAGTTGAATG* |
| 12 | *rs4830542* | *ACGTTGGATGAGGATGAGCTCATGCACAAG* | *ACGTTGGATGCAAAAATAAGAAGAAGAAAGG* | *TCTTTCTGGTCTCCCTCT* |
| 13 | *rs6632677* | *ACGTTGGATGAGCCTCAGAAGAGACCATAG* | *ACGTTGGATGAGTTCAGCTGGATCTTCTGC* | *CTCTACCATAGCTCTAGCCA* |
| 14 | *rs879922* | *ACGTTGGATGGCTCCAGCAAATTCAAGGAC* | *ACGTTGGATGGGCAGTTTATTGTACATTGTG* | *CTCAAGGACTGGGGTTA* |

Table S2 Descriptive information on ACE2 SNPs in study participants

| **ACE2 SNPs** | **Gene Region** | **MAF in CHB/CEU*** | **Genotype** | **Frequencies**  **(N/%)** | ***P_HWE_*-value^#^** |
| --- | --- | --- | --- | --- | --- |
| *rs1978124* | Intron 1 | 0.006/0.483 | *CC* | 292(77.7) | 0.003 |
|  |  |  | *CT* | 66(17.6) |  |
|  |  |  | *TT* | 18(4.8) |  |
| *rs2048683* | Intron 4 | 0.006/0.409 | *GG* | 316(84.0) | 0.003 |
|  |  |  | *GT* | 48(12.8) |  |
|  |  |  | *TT* | 12(3.2) |  |
| *rs2074192* | Intron 16 | 0.463/0.349 | *CC* | 135(35.9) | 0.659 |
|  |  |  | *CT* | 184(48.9) |  |
|  |  |  | *TT* | 57(15.2) |  |
| *rs2106809* | Intron 1 | 0.481/0.262 | *CC* | 59(15.7) | 0.011 |
|  |  |  | *CT* | 150(39.9) |  |
|  |  |  | *TT* | 167(44.4) |  |
| *rs2285666* | Intron 3 | 0.488/0.262 | *CC* | 97(25.8) | 0.303 |
|  |  |  | *CT* | 178(47.3) |  |
|  |  |  | *TT* | 101(26.9) |  |
| *rs233575* | Intron 16 | 0.006/0.362 | *CC* | 9(2.4) | 0.006 |
|  |  |  | *CT* | 60(16.0) |  |
|  |  |  | *TT* | 307(81.6) |  |
| *rs4240157* | Intron 14 | 0.044/0.383 | *CC* | 11(2.9) | 0.456 |
|  |  |  | *CT* | 95(25.3) |  |
|  |  |  | *TT* | 270(71.8) |  |
| *rs4646142* | Intron 7 | 0.488/0.261 | *CC* | 101(26.9) | 0.216 |
|  |  |  | *CG* | 176(46.8) |  |
|  |  |  | *GG* | 99(26.3) |  |
| *rs4646155* | Intron 8 | 0.038/0.000 | *CC* | 317(84.3) | <0.001 |
|  |  |  | *CT* | 18(4.8) |  |
|  |  |  | *TT* | 41(10.9) |  |
| *rs4646156* | Intron 8 | 0.006/0.403 | *AA* | 9(2.4) | 0.001 |
|  |  |  | *AT* | 54(14.4) |  |
|  |  |  | *TT* | 313(83.2) |  |
| *rs4646188* | Intron 7 | 0.000/0.087 | *CC* | 35(9.3) | <0.001 |
|  |  |  | *CT* | 53(14.1) |  |
|  |  |  | *TT* | 288(76.6) |  |
| *rs4830542* | 3’UTR | 0.044/0.383 | *CC* | 11(2.9) | 0.380 |
|  |  |  | *CT* | 93(24.7) |  |
|  |  |  | *TT* | 272(72.3) |  |
| *rs6632677* | Intron 1 | 0.094/0.007 | *CC* | 3(0.8) | 0.016 |
|  |  |  | *CG* | 30(8.0) |  |
|  |  |  | *GG* | 343(91.2) |  |
| *rs879922* | Intron 11 | 0.044/0.389 | *CC* | 11(2.9) | 0.146 |
|  |  |  | *CG* | 131(24.8) |  |
|  |  |  | *GG* | 234(62.2) |  |

*MAF: minor allele frequency; CHB：Han Chinese in Beijing, China；CEU：Utah residents with Northern and Western European ancestry

# *P*_HWE_ value for female participants (Hardy-Weinberg equilibrium)

Table S3 Baseline characteristics of study participants

|  | **Normotensive** | **Hypertensive** | ***P*-value** |
| --- | --- | --- | --- |
| N | 233 | 402 | - |
| Han：Uygur | 116:117 | 222:180 | 0.186 |
| Male：Female | 93:140 | 166:236 | 0.733 |
| Age(Y) | 57.7±12.6 | 58.8±10.9 | 0.298 |
| Smoking (%) | 43(18.3) | 82(20.4) | 0.553 |
| Drinking (%) | 40(17.2) | 83(20.6) | 0.285 |
| SBP(mmHg) | 116.2±9.5 | 155.8±13.3 | <0.001 |
| DBP(mmHg) | 73.9±7.4 | 85.5±13.5 | <0.001 |
| BMI(Kg/m^2^) | 23.5±4.6 | 26.1±4.1 | <0.001 |
| TRIG (mmol/L) | 1.29±0.95 | 1.35±0.63 | 0.350 |
| TC (mmol/L) | 4.56±1.12 | 4.64±1.21 | 0.370 |
| HDL-C(mmol/L) | 1.24±0.41 | 1.20±0.28 | 0.088 |
| LDL-C(mmol/L) | 2.38±0.76 | 2.86±0.80 | <0.001 |
| Lp(a)(g/L) | 0.24±0.19 | 0.24±0.21 | 0.621 |
| ApoA1/ApoB | 1.32±0.42 | 1.38±0.55 | 0.105 |
| FBG(mmol/L) | 5.90±1.89 | 6.02±1.92 | 0.422 |
| Cr(μmol/L) | 74.5±24.0 | 77.7±23.2 | 0.103 |
| BUN(mmol/L) | 5.20±1.99 | 5.28±1.72 | 0.614 |
| UA(μmol/L) | 308.5±135.7 | 355.4±131.7 | <0.001 |
| ALT(U/L) | 25.3±15.0 | 26.8±22.1 | 0.347 |
| AST(U/L) | 23.3±12.2 | 23.3±13.7 | 0.965 |
| Alb(g/L) | 37.2±4.4 | 37.6±6.0 | 0.412 |
| Na^+^(mmol/L) | 138.1±3.4 | 140.3±4.3 | <0.001 |
| Ca^2+^(mmol/L) | 2.19±0.11 | 2.20±0.12 | 0.096 |
| K^+^(mmol/L) | 4.03±0.41 | 3.97±0.49 | 0.097 |
| Mg^2+^(mmol/L) | 1.01±0.18 | 1.00±0.18 | 0.511 |
| HsCRP(mg/L) | 10.8±18.9 | 14.9±24.1 | 0.018 |
| ACE(U/L) | 36.6±19.8 | 42.0±25.7 | 0.006 |
| Renin(pg/mL) | 24.5±28.1 | 34.7±33.3 | <0.001 |
| Ang I(ng/L) | 2.07±1.44 | 2.70±1.77 | <0.001 |
| Ang II(ng/L) | 62.7±93.9 | 90.6±123.9 | 0.001 |
| ALD(ng/L) | 187.2±114.4 | 247.0±133.0 | <0.001 |

Table S4 Association of 7 ACE2 SNPs with EH in participants

| **ACE2 SNPs** | | **Normotensive**  **(N/%)** | **Hypertensive**  **(N/%)** | **OR(95%CI)*** | ***P*-value** |
| --- | --- | --- | --- | --- | --- |
| *rs1978124* | *CC* | 191(82.0) | 336(83.6) | 1.00 |  |
|  | *TT+CT* | 42(18.0) | 66(16.4) | 0.98(0.55-1.73) | 0.939 |
| *rs2048683* | *GG* | 203(87.1) | 357(88.8) | 1.00 |  |
|  | *TT+GT* | 30(12.9) | 45(11.2) | 0.85(0.46-1.58) | 0.601 |
| *rs2285666* | *CC* | 85(36.5) | 146(36.3) | 1.05(0.70-1.57) | 0.829 |
|  | *TT+CT* | 148(63.5) | 256(63.7) | 1.00 |  |
| *rs233575* | *CC+CT* | 36(15.5) | 48(11.9) | 1.00 |  |
|  | *TT* | 197(84.5) | 354(88.1) | 1.31(0.70-2.45) | 0.398 |
| *rs4646142* | *CC+CG* | 149(63.9) | 254(63.2) | 1.00 |  |
|  | *GG* | 84(36.1) | 148(36.8) | 1.07(0.72-1.60) | 0.736 |
| *rs4646156* | *AA+AT* | 30(12.9) | 48(11.9) | 1.00 |  |
|  | *TT* | 203(87.1) | 354(88.1) | 1.13(0.61-2.09) | 0.704 |
| *rs6632677* | *CC+CG* | 15(6.4) | 34(8.5) | 1.00 |  |
|  | *GG* | 218(93.6) | 368(91.5) | 0.79(0.39-1.58) | 0.502 |

* After adjustment for nationality, gender, age, smoking, BMI, TRIG, LDL-C, HDL-C, Lp(a), FBS, UA, HsCRP and Ang II

Table S5 Association of ACE2 SNPs with increased LDL-C (≥1.8mmol/L) in study subjects

| **ACE2 SNPs** | | **LDL-C<1.8mmol/L**  **(N/%)** | **LDL-C**≥**1.8mmol/L**  **(N/%)** | ***χ^2^*** | **P-value** | **OR(95%CI)*** | ***P*-value*** |
| --- | --- | --- | --- | --- | --- | --- | --- |
| *rs2048683* | *GG* | 131(97.8) | 429(85.6) | 14.940 | 0.000 | - | - |
|  | *TT+GT* | 3(2.2) | 72(14.4) |  |  |  |  |
| *rs2074192* | *CC* | 63(47.0) | 217(43.3) | 0.588 | 0.433 | 1.00 |  |
|  | *TT+CT* | 71(53.0) | 284(56.7) |  |  | 1.12(0.75-1.67) | 0.597 |
| *rs2285666* | *CC* | 44(32.8) | 187(37.3) | 0.921 | 0.337 | 1.37(0.89-2.12) | 0.148 |
|  | *TT+CT* | 90(67.2) | 314(62.7) |  |  | 1.00 |  |
| *rs4240157* | *CC+CT* | 23(17.2) | 112(22.4) | 1.702 | 0.192 | 1.24(0.70-2.19) | 0.464 |
|  | *TT* | 111(82.8) | 389(77.6) |  |  | 1.00 |  |
| *rs4646142* | *CC+CG* | 90(67.2) | 313(62.5) | 1.003 | 0.317 | 1.00 |  |
|  | *GG* | 44(32.8) | 188(37.5) |  |  | 1.37(0.89-2.10) | 0.152 |
| *rs4646155* | *CC* | 115(85.8) | 430(85.8) | 0.000 | 0.998 | 1.00 |  |
|  | *TT+CT* | 19(14.2) | 71(15.2) |  |  | 0.92(0.51-1.64) | 0.771 |
| *rs4646156* | *AA+AT* | 3(2.2) | 75(15.0) | 15.904 | 0.000 | - | - |
|  | *TT* | 131(97.8) | 426(85.0) |  |  |  |  |
| *rs4830542* | *CC+CT* | 23(17.2) | 110(22.0) | 1.466 | 0.226 | 1.20(0.67-2.13) | 0.539 |
|  | *TT* | 111(82.8) | 391(78.0) |  |  | 1.00 |  |
| *rs6632677* | *CC+CG* | 10(7.5) | 39(7.8) | 0.015 | 0.901 | 1.00 |  |
|  | *GG* | 124(92.5) | 462(92.2) |  |  | 0.88(0.42-1.83) | 0.729 |

* After adjustment for nationality, gender, age, BMI, EH, FBS, HsCRP and Ang II.

Table S6 Association of ACE2 SNPs with decreased HDL-C (<1.0mmol/L) in study subjects

| **ACE2 SNPs** | | **HDL-C≥1.0mmol/L**  **(N/%)** | **HDL-C<1.0mmol/L**  **(N/%)** | ***χ^2^*** | **P-value** | **OR(95%CI)*** | ***P*-value*** |
| --- | --- | --- | --- | --- | --- | --- | --- |
| *rs1978124* | *CC* | 406(79.5) | 121(97.6) | 23.233 | 0.000 | - | - |
|  | *TT+CT* | 105(20.5) | 3(2.4) |  |  |  |  |
| *rs2048683* | *CC* | 436(85.3) | 124(100.0) | 20.637 | 0.000 | - | - |
|  | *TT+CT* | 75(14.7) | 0(0.0) |  |  |  |  |
| *rs2074192* | *GG* | 220(43.1) | 60(48.4) | 1.152 | 0.283 | 0.95(0.60-1.50) | 0.832 |
|  | *TT+GT* | 291(56.9) | 64(51.6) |  |  | 1.00 |  |
| *rs233575* | *CC+CT* | 84(16.4) | 0(0.0) | 23.491 | 0.000 | - | - |
|  | *TT* | 427(83.6) | 124(100.0) |  |  |  |  |
| *rs4240157* | *CC+CT* | 128(25.0) | 7(5.6) | 22.443 | 0.000 | 1.12(0.42-3.01) | 0.820 |
|  | *TT* | 383(75.0) | 117(94.4) |  |  | 1.00 |  |
| *rs4646156* | *AA+AT* | 78(15.3) | 0(0.0) | 21.578 | 0.00 | - | - |
|  | *TT* | 433(84.7) | 124(100.0) |  |  |  |  |
| *rs4830542* | *CC+CT* | 126(24.7) | 7(5.6) | 21.784 | 0.000 | 1.00 |  |
|  | *TT* | 385(75.3) | 117(94.4) |  |  | 0.75(0.27-2.09) | 0.580 |
| *rs6632677* | *CC+CG* | 43(8.4) | 6(4.8) | 1.792 | 0.181 | 1.00 |  |
|  | *GG* | 468(91.6) | 118(95.2) |  |  | 1.57(0.57-3.94) | 0.408 |
| *rs879922* | *CC+CG* | 164(32.1) | 7(5.6) | 35.474 | 0.000 | 1.00 |  |
|  | *GG* | 347(67.9) | 117(94.4) |  |  | 1.06(0.39-2.89) | 0.905 |

* After adjustment for nationality, gender, age, BMI, EH, FBS, HsCRP and Ang II.

Table S7 Association of ACE2 SNPs with increased TC (≥5.2mmol/L) in study subjects

| **ACE2 SNPs** | | **TC<5.2mmol/L**  **(N/%)** | **TC≥5.2mmol/L**  **(N/%)** | ***χ^2^*** | **P-value** | **OR(95%CI)*** | ***P*-value*** |
| --- | --- | --- | --- | --- | --- | --- | --- |
| *rs1978124* | *CC* | 435(83.3) | 92(81.4) | 0.242 | 0.623 | 1.56(0.86-2.84) | 0.145 |
|  | *TT+CT* | 87(16.7) | 21(18.6) |  |  | 1.00 |  |
| *rs2048683* | *GG* | 465(89.1) | 95(84.1) | 2.238 | 0.135 | 1.00 |  |
|  | *TT+GT* | 57(10.9) | 18(15.9) |  |  | 0.98(0.52-1.84) | 0.943 |
| *rs2106809* | *CC+CT* | 247(47.3) | 49(43.4) | 0.584 | 0.445 | 1.00 |  |
|  | *TT* | 275(52.7) | 64(56.6) |  |  | 1.09(0.68-1.75) | 0.709 |
| *rs2285666* | *CC* | 190(36.4) | 41(36.3) | 0.001 | 0.982 | 1.00 |  |
|  | *TT+CT* | 333(63.6) | 72(63.7) |  |  | 0.97(0.61-1.53) | 0.884 |
| *rs233575* | *CC+CT* | 63(12.1) | 21(18.6) | 3.435 | 0.064 | 1.02(0.56-1.89) | 0.939 |
|  | *TT* | 459(87.9) | 92(81.4) |  |  | 1.00 |  |
| *rs4646142* | *CC+CG* | 331(63.4) | 72(63.7) | 0.004 | 0.951 | 1.04(0.61-1.77) | 0.895 |
|  | *GG* | 191(36.6) | 41(36.3) |  |  | 1.00 |  |
| *rs4646155* | *CC* | 436(83.5) | 109(96.5) | 12.777 | 0.000 | - | - |
|  | *TT+CT* | 86(16.5) | 4(3.5) |  |  |  |  |
| *rs6632677* | *CC+CG* | 43(8.2) | 6(5.3) | 1.118 | 0.290 | 1.00 |  |
|  | *GG* | 479(91.8) | 107(94.7) |  |  | 1.75(0.71-4.32) | 0.226 |

* After adjustment for nationality, gender, age, BMI, EH, FBS, HsCRP and Ang II.

Table S8 Association of ACE2 SNPs with increased TRIG (≥1.7mmol/L) in study subjects

| **ACE2 SNPs** | | **TRIG<1.7mmol/L**  **(N/%)** | **TRIG≥1.7mmol/L**  **(N/%)** | ***χ^2^*** | **P-value** | **OR(95%CI)*** | ***P*-value*** |
| --- | --- | --- | --- | --- | --- | --- | --- |
| *rs1978124* | *CC* | 418(83.3) | 109(82.0) | 0.128 | 0.720 | 1.09(0.61-1.95) | 0.768 |
|  | *TT+CT* | 84(16.7) | 24(18.0) |  |  | 1.00 |  |
| *rs2048683* | *GG* | 442(88.0) | 118(88.7) | 0.046 | 0.830 | 1.22(0.63-2.34) | 0.561 |
|  | *TT+GT* | 60(12.0) | 15(11.3) |  |  | 1.00 |  |
| *rs2074192* | *GG* | 228(45.4) | 52(39.1) | 1.704 | 0.192 | 1.00 |  |
|  | *TT+GT* | 274(54.6) | 81(60.9) |  |  | 1.29(0.86-1.94) | 0.212 |
| *rs2285666* | *CC* | 179(35.7) | 52(39.1) | 0.538 | 0.463 | 1.11(0.72-1.69) | 0.645 |
|  | *TT+CT* | 323(64.3) | 81(60.9) |  |  | 1.00 |  |
| *rs233575* | *CC+CT* | 66(13.1) | 18(13.5) | 0.014 | 0.907 | 1.00 |  |
|  | *TT* | 436(86.9) | 115(86.5) |  |  | 1.12(0.60-2.10) | 0.722 |
| *rs4240157* | *CC+CT* | 102(20.3) | 33(24.8) | 1.268 | 0.260 | 1.09(0.65-1.84) | 0.736 |
|  | *TT* | 400(79.7) | 100(75.2) |  |  | 1.00 |  |
| *rs4646142* | *CC+CG* | 319(63.5) | 84(63.2) | 0.007 | 0.934 | 1.05(0.68-1.61) | 0.830 |
|  | *GG* | 183(36.5) | 49(36.8) |  |  | 1.00 |  |
| *rs4646155* | *CC* | 427(85.1) | 118(88.7) | 1.159 | 0.282 | 1.43(0.76-2.69) | 0.270 |
|  | *TT+CT* | 75(14.9) | 15(11.3) |  |  | 1.00 |  |
| *rs4646156* | *AA+AT* | 63(12.5) | 15(11.3) | 0.158 | 0.691 | 1.00 |  |
|  | *TT* | 439(87.5) | 118(88.7) |  |  | 1.32(0.68-2.53) | 0.411 |
| *rs4830542* | *CC+CT* | 100(19.9) | 33(24.8) | 1.519 | 0.218 | 1.12(0.66-1.89) | 0.670 |
|  | *TT* | 402(80.1) | 100(75.2) |  |  | 1.00 |  |
| *rs6632677* | *CC+CG* | 44(8.8) | 5(3.8) | 3.699 | 0.054 | - | - |
|  | *GG* | 458(91.2) | 128(96.2) |  |  |  |  |
| *rs879922* | *CC+CG* | 129(25.7) | 42(31.6) | 1.849 | 0.174 | 1.13(0.66-1.93) | 0.658 |
|  | *GG* | 373(74.3) | 91(68.4) |  |  | 1.00 |  |

* After adjustment for nationality, gender, age, BMI, EH, FBS, HsCRP and Ang II.

**References**

1. Carey RM, Whelton PK, Committee AAHGW: **Prevention, Detection, Evaluation, and Management of High Blood Pressure in Adults: Synopsis of the 2017 American College of Cardiology/American Heart Association Hypertension Guideline.** *Ann Intern Med* 2018, **168:**351-358.

2. Zhou YF, Yan H, Hou XP, Miao JL, Zhang J, Yin QX, Li JJ, Zhang XY, Li YY, Luo HL: **Association study of angiotensin converting enzyme gene polymorphism with elderly diabetic hypertension and lipids levels.** *Lipids Health Dis* 2013, **12:**187.

3. Kernan WN, Ovbiagele B, Black HR, Bravata DM, Chimowitz MI, Ezekowitz MD, Fang MC, Fisher M, Furie KL, Heck DV, et al: **Guidelines for the prevention of stroke in patients with stroke and transient ischemic attack: a guideline for healthcare professionals from the American Heart Association/American Stroke Association.** *Stroke* 2014, **45:**2160-2236.

4. Wang W, Li L, Zhou Z, Gao J, Sun Y: **Effect of spironolactone combined with angiotensin-converting enzyme inhibitors and/or angiotensin II receptor blockers on chronic glomerular disease.** *Exp Ther Med* 2013, **6:**1527-1531.
